# A dose-finding study of raltitrexed (tomudex) with cisplatin and epirubicin in advanced gastro-oesophageal adenocarcinoma

**DOI:** 10.1054/bjoc.2000.1165

**Published:** 2000-05-18

**Authors:** M M Eatock, D A Anthony, M El-Abassi, P Wilson, J Paul, M Smith, M Soukop, T R J Evans

**Affiliations:** CRC Department of Medical Oncology, Beatson Oncology Centre, Western Infirmary, Dumbarton Road, Glasgow, G11 6NT, UK; Department of Radiology, Glasgow Royal Infirmary, Castle Street, Glasgow, G4 0SF, UK; Zeneca Pharmaceuticals, Alderley Park, Macclesfield, SK10 4TG, UK; Department of Medical Oncology, Glasgow Royal Infirmary, Castle Street, Glasgow, G4 0SF, UK

**Keywords:** gastro-oesophageal cancer, epirubicin, cisplatin, tomudex, chemotherapy

## Abstract

The standard treatment for advanced gastro-oesophageal cancer in the UK is epirubicin, cisplatin and continuous infusion 5-fluoruracil by an indwelling central venous catheter (ECF), which has significant morbidity. Raltitrexed (tomudex), a specific inhibitor of thymidylate synthase with a long plasma terminal half-life (50–100 h) has activity in gastro-intestinal tract malignancy. To reduce the Hickman line-associated morbidity of ECF; we have conducted a dose-finding study of tomudex combined with epirubicin and cisplatin. Twenty-four patients (22 males, two female), median age 63 years (range 21–75), ECOG performance status ≤ 2 with histologically proven, unresectable or metastatic gastric (14 patients), gastro-oesophageal junction (nine patients) or oesophageal (one patient) adenocarcinoma received treatment with 3-weekly cisplatin 60 mg m^−2^, epirubicin 50 mg m^−2^and tomudex at doses of 2 mg m^−2^, 2.5 mg m^−2^or 3 mg m^−2^in successive cohorts. Six patients were treated per dose level with no intra-patient dose escalation. Dose escalation occurred after six patients had completed at least one cycle of chemotherapy at the previous dose level. After defining the maximum tolerated dose a further six patients were treated at the preceding dose level to assess toxicity at the proposed phase II dose. A total of 102 cycles (50% completed 6 cycles) were administered. The dose-limiting toxicities are neutropenia and diarrhoea occurring in 2/6 patients at the 3 mg m^−2^dose level. Of those patients evaluable for response, there were eight partial and one complete response (overall response rate 38%). The median survival was 9.9 months. ECT is an active regimen in oesophagogastric adenocarcinoma. The recommended dose of tomudex for further study in combination with epirubicin and cisplatin is 2.5 mg m^−2^. © 2000 Cancer Research Campaign


British Journal of Cancer (2000) 82(12), 1925–1931
© 2000 Cancer Research Campaign
doi: 10.1054/ bjoc.2000.1165, available online at http://www.idealibrary.com on IDEAL®

### A dose-finding study of raltitrexed (tomudex) with cisplatin and epirubicin in advanced gastro-oesophageal adenocarcinoma

**MM Eatock[1], DA Anthony[1], M El-Abassi[2], P Wilson[1], J Paul[1], M Smith[3], M Soukop[4] and TRJ Evans[1]**

[1]CRC Department of Medical Oncology, Beatson Oncology Centre, Western Infirmary, Dumbarton Road, Glasgow G11 6NT, UK; [2]Department of Radiology, Glasgow Royal Infirmary, Castle Street, Glasgow G4 0SF, UK; [3]Zeneca Pharmaceuticals, Alderley Park, Macclesfield SK10 4TG, UK; [4]Department of Medical Oncology, Glasgow Royal Infirmary, Castle Street, Glasgow G4 0SF, UK;

**Summary** The standard treatment for advanced gastro-oesophageal cancer in the UK is epirubicin, cisplatin and continuous infusion 5-fluoruracil by an indwelling central venous catheter (ECF), which has significant morbidity. Raltitrexed (tomudex), a specific inhibitor of thymidylate synthase with a long plasma terminal half-life (50–100 h) has activity in gastro-intestinal tract malignancy. To reduce the Hickman line-associated morbidity of ECF; we have conducted a dose-finding study of tomudex combined with epirubicin and cisplatin. Twenty-four patients (22 males, two female), median age 63 years (range 21–75), ECOG performance status ≤ 2 with histologically proven, unresectable or metastatic gastric (14 patients), gastro-oesophageal junction (nine patients) or oesophageal (one patient) adenocarcinoma received treatment with 3-weekly cisplatin 60 mg $m^{-2}$, epirubicin 50 mg $m^{-2}$ and tomudex at doses of 2 mg $m^{-2}$, 2.5 mg $m^{-2}$ or 3 mg $m^{-2}$ in successive cohorts. Six patients were treated per dose level with no intra-patient dose escalation. Dose escalation occurred after six patients had completed at least one cycle of chemotherapy at the previous dose level. After defining the maximum tolerated dose a further six patients were treated at the preceding dose level to assess toxicity at the proposed phase II dose. A total of 102 cycles (50% completed 6 cycles) were administered. The dose-limiting toxicities are neutropenia and diarrhoea occurring in 2/6 patients at the 3 mg $m^{-2}$ dose level. Of those patients evaluable for response, there were eight partial and one complete response (overall response rate 38%). The median survival was 9.9 months. ECT is an active regimen in oesophagogastric adenocarcinoma. The recommended dose of tomudex for further study in combination with epirubicin and cisplatin is 2.5 mg $m^{-2}$. © 2000 Cancer Research Campaign

**Keywords:** gastro-oesophageal cancer; epirubicin; cisplatin; tomudex; chemotherapy

Gastric adenocarcinoma has a poor prognosis, accounting for 10 000 deaths per year in the UK. Although its incidence is declining, it remains the second most common tumour world-wide and the fourth commonest cancer in Europe. At presentation around 30% of patients have metastatic disease and a further 30% have locally advanced disease penetrating the serosal surface of the stomach or directly invading adjacent organs. With locally advanced disease, curative surgery is only possible in a minority of patients and recurrent disease affects 80% of patients within 5 years of potentially curative surgery. Therefore the prognosis remains poor with an overall survival of around 20% at 5 years (Allum et al, 1989).

Combination chemotherapy results in a significant survival advantage in patients with advanced gastric cancer compared with best supportive care in randomized clinical trials. High response rates may be obtained with the use of protracted venous infusional 5-fluorouracil (5-FU), cisplatin and epirubicin – the ECF regimen (Findlay et al, 1994). In an initial study with this regimen, an overall response rate of 71% and a complete response rate of 12% were observed. These encouraging results have been confirmed in two subsequent studies, with overall response rates of around 60% and with complete responses occurring in around 10% of patients (Highley et al, 1994; Zaniboni et al, 1995). In a multi-centre randomized study, ECF resulted in significantly better response rate (45%) and median survival (8.9 months), with significantly less toxicity compared to the FAMtx regimen (Webb et al, 1997). Consequently the ECF regimen is considered the treatment of choice for advanced adenocarcinoma of the oesophagus and stomach by many clinicians in the UK.

This regimen, however, requires protracted venous infusion of 5-FU through a Hickman catheter, which is associated with significant morbidity. The most common problems encountered are thrombosis and sepsis requiring line removal in up to 15% of patients (Webb et al, 1997). Consequently, replacing the continuous infusion of 5-FU by a drug with a related mechanism of action would overcome the need for inserting a Hickman catheter and its associated morbidity. As inhibition of thymidylate synthase (TS) is an important mechanism of action of infusional 5-FU, one such strategy is to combine epirubicin and cisplatin with a specific TS inhibitor. TS catalyses the reductive methylation of deoxyuridylate (dUMP) to thymidylate (TMP), the rate-limiting step in the synthesis of deoxythmidine triphosphate (TTP). TTP is the only nucleotide specifically required for DNA synthesis and has been postulated as an ideal target for anticancer drugs.

Raltitrexed (tomudex) is a quinazolone-based, water-soluble anti-folate, which is extensively and efficiently poly-glutamated. The poly-glutamates are 100 times more potent inhibitors of TS than the parent drug and are retained intra-cellularly allowing a

*Received 24 May 1999*
*Revised 11 February 2000*
*Accepted 17 February 2000*

*Correspondence to:* MM Eatock convenient 3-weekly dosing schedule. The maximum-tolerated dose of tomudex is 3.5 mg $m^{-2}$, resulting in severe malaise, nausea, asthenia and anti-proliferative toxicity including bone marrow and gastrointestinal toxicity (Clarke et al, 1996). Consequently the recommended dose for phase II studies was 3 mg $m^{-2}$. In these studies, raltitrexed has demonstrated single-agent activity in colorectal (Jackman et al, 1995), breast (Smith et al, 1996), pancreatic (Pazdur et al, 1996) and ovarian carcinoma (Gore et al, 1995). In colorectal cancer, randomized trials have shown similar activity to 5-FU (Cunningham, 1998).

We have therefore conducted a dose-finding study in previously untreated patients with adenocarcinoma of the oesophagus and stomach to define the optimal dose of tomudex used in combination with epirubicin and cisplatin. This will form the basis of further studies to determine the activity of this regimen with the aim of avoiding the morbidity of Hickman catheter insertion for the administration of infusional 5-FU.

### PATIENTS AND METHODS

### Eligibility criteria

The Local Research Ethics Committee in each of the participating centres approved this study. All patients entered into this study had histologically confirmed adenocarcinoma of the stomach, oesophagus or gastro-oesophageal junction Inoperability was determined on the basis of clinical evaluation, radiological imaging, and laparoscopy or laparotomy with failed resection. Eligibility criteria included: estimated life expectancy ≥12 weeks; at least one site of bi-dimensionally measurable disease or evaluable disease; adequate renal function (calculated creatinine clearance ≥60 ml $min^{-1}$ by the Cockcroft and Gault formula); adequate hepatic function (serum bilirubin ≤1.5 times the upper limit of the reference range, transaminases <5 times the upper limit of the reference range) and adequate haematological function (haemoglobin ≥10 g $dl^{-1}$; neutrophil count $\geq 1.5 \times 10^9$ $l^{-1}$; platelets $\geq 100 \times 10^9$ $l^{-1}$ Patients who had received previous chemotherapy for advanced disease were excluded. Patients who had received neo-adjuvant or adjuvant chemotherapy with the ECF regimen were eligible if more than 1 year had elapsed from the completion of this treatment. Patient performance status was assessed using the ECOG criteria and those patients with performance status ≤2 were considered eligible for this study. All patients gave written informed consent prior to starting treatment.

### Chemotherapy

Cisplatin (60 mg $m^{-2}$) and epirubicin (50 mg $m^{-2}$) were administered on day 1 and then every 21 days for up to a maximum of six cycles of treatment. Cisplatin was administered as a short intravenous infusion with standard pre- and post-hydration protocols, magnesium and potassium supplementation and anti-emetics (dexamethasone and granisetron). Raltitrexed was administered as a short intravenous infusion over 15 min prior to the cisplatin on day 1 of a 3-weekly cycle. A maximum of six patients were treated at each of three pre-determined dose levels (2, 2.5 and 3 mg $m^{-2}$) and no intra-patient dose escalation was permitted. Dose escalation was performed after all six patients in the previous dose level had completed at least one cycle of chemotherapy.

### Evaluation of toxicity

Chemotherapy toxicity was graded using the NCIC-CTC expanded common toxicity criteria. Toxicity, performance status and biochemical profile were assessed prior to each chemotherapy cycle, and full blood count was performed weekly during all treatment cycles. Dose escalation and determination of dose-limiting toxicity (DLT) and maximum-tolerated dose (MTD) was on the basis of toxicity from the first cycle of chemotherapy only.

DLT was defined as grade IV or complicated grade III haematological toxicity (neutropenia with fever, thrombocytopenia with bleeding or bruising); grade III/IV non-haematological toxicity other than alopecia, nausea or vomiting; or stomatitis ≥ grade II. MTD was determined when at least two out of the six patients treated at that dose level experienced any DLT. Once the MTD was defined a further six patients were treated at one dose level below the MTD to gain further experience of the toxicity with this regimen. Cumulative toxicity was also recorded for all subsequent chemotherapy cycles at all dose levels.

### Dose modifications and delays

Dose modifications were performed on the basis of toxicity. Administration of all three agents was delayed until adequate haematological recovery (absolute neutrophil count $\geq 1.5 \times 10^9$ $l^{-1}$, platelets $\geq 100 \times 10^9$ $l^{-1}$) up to a maximum of 3 weeks. Dose modification of epirubicin was based on haematological parameters at the time that each chemotherapy cycle was due. In the event of clinically significant thrombocytopenia or an episode of neutropaenic sepsis, the dose of epirubicin was reduced by 25% for all subsequent cycles of chemotherapy even if haematological recovery had occurred at the time of treatment. If patients had uncomplicated grade IV neutropenia the epirubicin dose was maintained in subsequent cycles. Raltitrexed dose was not modified in the face of uncomplicated grade IV myelosuppression if this had recovered prior to day 21.

For non-haematological toxicity (other than alopecia, nausea and vomiting), raltitrexed was discontinued for grade IV toxicity and given at 75% of the starting dose for grade III toxicity respectively. Raltitrexed was given at full dose if the calculated creatinine clearance (CCC) was ≥ 60 ml $min^{-1}$, was reduced to 50% of the starting dose and given at 28-day intervals for CCC 25–59 ml $min^{-1}$ and was discontinued for CCC <25 ml $min^{-1}$.

Cisplatin dose modifications for impaired renal function were made according to previously published guidelines (Findlay et al, 1994). Cisplatin was administered at full dose if the CCC was ≥ 60 ml $min^{-1}$, and discontinued if CCC was < 40 ml $min^{-1}$. For CCC 40–59 ml $min^{-1}$ the total dose of cisplatin in milligrams administered was equivalent to the CCC (e.g. if CCC was 50 ml $min^{-1}$ the cisplatin dose was 50 mg). Following a dose reduction all subsequent doses were administered at the modified dose unless there was recovery of renal function to normal levels.

### Evaluation of response and overall survival

Pretreatment evaluations included a full medical history and examination, full blood count and biochemical profile including urea, electrolytes, calculated creatinine clearance and liver function tests. Computerized tomography (CT) scan of the abdomen and any other sites of disease as appropriate was performed prior to starting treatment and repeated after three and six cycles of

© 2000 Cancer Research Campaign

**Table 1** Patient characteristics on study entry

| Characteristic | Number |
|---|---|
| Total number of patients | 24 |
| M/F | 22/2 |
| Median age (years) | 63 |
| Range | 21–75 |
| Primary tumour | |
| Oesophagus | 1 (4%) |
| Gastro-oesophageal junction | 9 (38%) |
| Gastric | 14 (58%) |
| Histology | |
| Intestinal | 5 (21%) |
| Diffuse | 6 (25%) |
| Unknown | 13 (54%) |
| Stage II | 1 (4%) |
| Stage IIIA | 2 (8%) |
| Stage IIIB | 4 (17%) |
| Stage IV | 17 (71%) |
| Previous treatment | |
| None | 8 (33%) |
| Surgery alone | 13 (54%) |
| Surgery and XRT | 3 (13%) |
| ECOG performance status | |
| 0 | 10 (42%) |
| 1 | 9 (38%) |
| 2 | 5 (21%) |

chemotherapy to assess response. Endoscopy was also repeated after three and six cycles as response assessment in those with endoscopically evaluable disease. Evaluation of response was based on the standard WHO criteria. Subsequently CT scans and endoscopy were not performed routinely but at the investigator's discretion. The time to disease progression was recorded where known and overall survival curves were calculated using the Kaplan and Meier method.

### RESULTS

Twenty-four eligible patients received a total of 102 cycles of treatment between 1997 and 1998 at the Beatson Oncology Centre, Glasgow or at Glasgow Royal Infirmary. The patient characteristics at baseline are summarized in Table 1. Thirteen patients had previously been treated with surgery alone, and three with both surgery and radiotherapy. No patients had received previous neo-adjuvant or adjuvant chemotherapy. The median number of treatment cycles administered per patient was 5.5 (range 1–6) and 50% of the patients in this study completed six cycles of treatment.

### Toxicity (first cycle)

#### *Haematological toxicity*

Haematological toxicity following the first cycle of treatment is summarized in Tables 2 and 3. One patient, at the first dose level (2 mg $m^{-2}$ raltitrexed), had severe haematological toxicity with grade IV leukopenia, neutropenic sepsis, thrombocytopenia and renal failure secondary to hypotension as a result of gastrointestinal haemorrhage and sepsis. Gastrointestinal haemorrhage had been a problem prior to commencing chemotherapy in this patient and, after one cycle of chemotherapy, death occurred from recurrent gastrointestinal haemorrhage with thrombocytopenia and rapidly progressive disease.

In the second cohort (dose level 2.5 mg $m^{-2}$ raltitrexed), one patient had uncomplicated grade IV neutropenia after one cycle of

**Table 2** Haematological toxicity – first cycle only (nadir counts)

| Dose of Tomudex | Median nadir neutrophil count (range) | Median nadir platelet count (range) | Median nadir WCC (range) |
|---|---|---|---|
| 2 mg $m^{-2}$ (*n* = 6) | 0.69 (0.50–1.80) | 206.5 (4–271) | 2.24 (0.98–3.50) |
| 2.5 mg $m^{-2}$ (*n* = 12) | 1.40 (0.37–4.10) | 222 (160–441) | 3.22 (1.31–6.70) |
| 3 mg $m^{-2}$ (*n* = 6) | 1.46 (0.10–4.40) | 201 (89–411) | 2.97 (0.60–6.20) |

Median time to nadir = 14 days (range 7–21 days, interquartile range 12–15 days).

**Table 3** Haematological toxicity – first cycle only (NCI-CTC grading)

| | Grade | Tomudex 2.0 mg $m^{-2}$ (*n* = 6) n | Tomudex dose 2.5 mg $m^{-2}$ (*n* = 12) n | Tomudex 3.0 mg $m^{-2}$ (*n* = 6) n |
|---|---|---|---|---|
| | 2 | 2 | 3 | 0 |
| Neutropenia | 3 | 4 | 1 | 2 |
| | 4 | 0 | 2 | 1 |
| | 2 | 3 | 2 | 1 |
| Leucopenia | 3 | 1 | 2 | 1 |
| | 4 | 1 | 0 | 1 |
| | 2 | 0 | 0 | 0 |
| Thrombocytopenia | 3 | 0 | 0 | 0 |
| | 4 | 1 | 0 | 0 |
| | 2 | 1 | 4 | 2 |
| Anaemia | 3 | 1 | 0 | 0 |
| | 4 | 0 | 0 | 0 |

*© 2000 Cancer Research Campaign*

**Table 4** Non-haematological toxicity – first cycle only (NCI-CTC grading)

| | Grade | Tomudex 2.0 mg m$^{-2}$ (*n* = 6) n | Tomudex dose 2.5 mg m$^{-2}$ (*n* = 12) n | Tomudex 3.0 mg m$^{-2}$ (*n* = 6 n |
|---|---|---|---|---|
| | 2 | 1 | 1 | 2 |
| Nausea | 3 | 0 | 1 | 0 |
| | 2 | 0 | 3 | 0 |
| Vomiting | 3 | 0 | 0 | 0 |
| | 2 | 0 | 0 | 0 |
| Diarrhoea | 3 | 0 | 0 | 1 |
| | 4 | 1 | 0 | 1 |
| | 2 | 0 | 0 | 1 |
| Stomatitis | 3 | 0 | 0 | 0 |
| | 4 | 1 | 0 | 0 |
| | 2 | 0 | 0 | 1 |
| Anorexia | 3 | 0 | 0 | 0 |

**Table 5** Cumulative haematological toxicity – all cycles (NCI-CTC grading)

| | Grade | Dose level 1 Tomudex 2.0 mg m$^{-2}$ (*n* = 6) *n* | Dose level 2 Tomudex 2.5 mg m$^{-2}$ (*n* = 12) *n* | Dose level 3 Tomudex 3 mg m$^{-2}$ (*n* = 6) *n* |
|---|---|---|---|---|
| | 2 | 0 | 1 | 2 |
| Neutropenia | 3 | 5 | 4 | 1 |
| | 4 | 1 | 4 | 3 |
| | 2 | 4 | 5 | 2 |
| Leucopenia | 3 | 1 | 3 | 0 |
| | 4 | 1 | 0 | 3 |
| | 2 | 0 | 0 | 0 |
| Thrombocytopenia | 3 | 0 | 0 | 0 |
| | 4 | 1 | 0 | 0 |
| | 2 | 1 | 7 | 5 |
| Anaemia | 3 | 1 | 0 | 0 |
| | 4 | 1 | 0 | 0 |

treatment. In the third cohort (dose level 3.0 mg m$^{-2}$ raltitrexed) one patient developed complicated grade III and one patient complicated grade IV neutropenia.

### *Non-haematological toxicity*

Non-haematological toxicity following the first cycle of treatment is summarized in Table 4. In the first cohort, one patient developed grade IV diarrhoea and stomatitis after the first treatment (see below). In the third cohort of patients (raltitrexed 3.0 mg m$^{-2}$) two patients experienced grade III or IV diarrhoea after one cycle of treatment. Biochemical toxicity was mild and was not dose-limiting in any of the patient cohorts (infra vide).

### Toxicity (all other cycles)

### *Haematological toxicity*

The maximum haematological toxicity for all cycles of treatment and nadir blood counts are summarized in Table 5.

One patient at the first dose level had the fifth cycle of treatment delayed for neutropenia on day 21. In the second cohort one patient experienced grade IV uncomplicated neutropenia after three cycles of treatment. There were no delays in treatment in this group of patients for toxicity.

Two patients in the third cohort had neutropenic sepsis after three cycles of treatment. In the fourth cohort (2.5 mg m$^{-2}$) two further patients experienced uncomplicated grade IV neutropenia during their treatment.

### *Non-haematological toxicity*

Worst non-haematological toxicity for all administered cycles is shown in Table 6. Alopecia, as expected was common. Nausea and vomiting was well controlled with serotonin antagonists, except for three patients who had grade III nausea and vomiting. Dose-limiting non-haematological toxicity occurring after the first cycle of treatment occurred in two patients treated with 3 mg m$^{-2}$ of raltitrexed (diarrhoea and stomatitis in one patient, diarrhoea alone in one patient). At the expanded dose level of 2.5 mg m$^{-2}$, one patient experienced grade IV diarrhoea after three cycles of treatment.

### *Biochemistry*

***Hepatic function*** One patient in the first cohort developed grade I reversible transaminitis, 8 days after the first and second cycles of chemotherapy. This did not recur with subsequent treatments. One patient in the second cohort developed grade II transaminitis following one cycle of treatment. This occurred 8 days after treatment and returned to normal on day 13 and was felt to be drug-related. In the third cohort, one patient had grade I and one patient grade II transaminitis, which was probably drug-related and

 © 2000 Cancer Research Campaign

**Table 6** Cumulative non-haematological toxicity – all cycles (NCI-CTC grading).

| | Grade | Dose level 1 Tomudex 2.0 mg m$^{-2}$ (*n* = 6) *n* | Dose level 2 Tomudex 2.5 mg m$^{-2}$ (*n* = 12) *n* | Dose level 3 Tomudex 3.0 mg m$^{-2}$ (*n* = 6) *n* |
|---|---|---|---|---|
| Alopecia | 3 | 6 | 8 | 4 |
| | 2 | 1 | 2 | 4 |
| Nausea | 3 | 1 | 1 | 0 |
| | 2 | 2 | 2 | 1 |
| Vomiting | 3 | 0 | 1 | 0 |
| | 2 | 1 | 1 | 0 |
| Diarrhoea | 3 | 0 | 0 | 1 |
| | 4 | 1 | 1 | 3 |
| | 2 | 0 | 0 | 1 |
| Stomatitis | 3 | 0 | 0 | 0 |
| | 4 | 1 | 0 | 0 |
| Neutropaenic sepsis | | 1 | 0 | 2 |
| | 2 | 0 | 1 | 2 |
| Anorexia | 3 | 0 | 0 | 1 |

resolved spontaneously. A further patient in the third cohort developed grade I transaminitis attributed to disease progression.

***Renal function*** There was no evidence of renal toxicity due to chemotherapy in any cohort. In the first cohort, one patient developed renal failure as a result of sepsis. At the second dose level, one patient developed a transient rise in serum creatinine 11 days following the first cycle of treatment, which returned to normal after 5 days. One patient at the first dose level (raltitrexed 2 mg m$^{-2}$) had a dose reduction in tomudex and cisplatin due to a fall in calculated creatinine clearance, which subsequently returned to normal. One patient treated with a dose of 2.5 mg m$^{-2}$ of tomudex had a dose reduction of both cisplatin and tomudex for renal impairment on the third cycle, following which chemotherapy was discontinued due to disease progression.

### DLT and MTD

The DLTs were neutropaenia, diarrhoea and stomatitis occurring in two patients at the third dose level (3 mg m$^{-2}$ raltitrexed) after one cycle of treatment. The MTD of raltitrexed in this combination was therefore defined as 3 mg/m$^2$. Grade IV neutropenia occurred in one patient after one cycle and a further two patients after three cycles of chemotherapy. This was complicated by sepsis in two of these patients and as a result two subsequent patients at this dose level received a reduced raltitrexed dose of 2.5 mg m$^{-2}$ after completing the first cycle of treatment.

Two patients died during this study. The first patient was a 71-year-old man with a gastric cancer, who was admitted to hospital 6 days following treatment at the first tomudex dose level with NCI-CTC grade 3 neutropenia, grade 4 neutropenic sepsis and gastrointestinal haemorrhage. Renal failure occurred as a result of the gastrointestinal haemorrhage and neutropenic sepsis and, despite treatment with intravenous fluid resuscitation, intravenous antibiotic therapy and transfusions of platelets and red cell concentrates, he died 4 days following hospital admission. Gastrointestinal bleeding, which had been present prior to treatment, was considered to have contributed significantly to his death. This was exacerbated by myelosuppression, thrombocytopenia, and neutropenic sepsis, and therefore toxicity from chemotherapy also contributed significantly to this patient's death, and was the primary cause of death.

The second patient was treated with 3 mg m$^{-2}$ of tomudex. Eight days following the first cycle of treatment she was admitted with NCI-CTC grade 4 neutropenia with grade 4 diarrhoea, which required intravenous fluid resuscitation. Twelve days following treatment she developed grade 4 neutropenic sepsis. Blood cultures were positive for *Streptococcus pneumoniae* and she was treated with appropriate intravenous antibiotics. The fever resolved after 48 h. Her neutrophil count had recovered to normal within 6 days of admission to hospital. She then developed progressive abdominal distension and clinical signs consistent with a large bowel obstruction, which was confirmed on barium studies. This was considered to be due to disease progression. Her condition deteriorated and she developed a marked metabolic acidosis with normal renal function. Lactic acidosis may have been present, however serum lactate was not checked. She died 29 days following the administration of chemotherapy. The exact cause of death was not known and post-mortem examination was refused. Death was not considered to be due directly to toxicity of chemotherapy.

Two further patients treated with 3 mg m$^{-2}$ of tomudex developed grade 4 diarrhoea along with grade 3 or 4 neutropenia. One of these patients developed this after one cycle of treatment and the second after three cycles. Both patients recovered fully after 10 and 8 days respectively following intravenous fluid resuscitation and anti-diarrhoeal therapy with loperamide.

### Response and survival

Of the 24 patients enrolled in the study, 20 patients completed at least three cycles of therapy and were eligible for response assessment. One further patient had clinically progressive disease after one cycle of treatment and has been included in the assessment of response. One patient had a complete response and eight patients had a partial response to treatment. Six patients had stable disease, one of these patients, however, had a good endoscopic response to treatment. Five patients had radiologically progressive disease after three cycles of treatment. The overall response rate was 38%

© 2000 Cancer Research Campaign

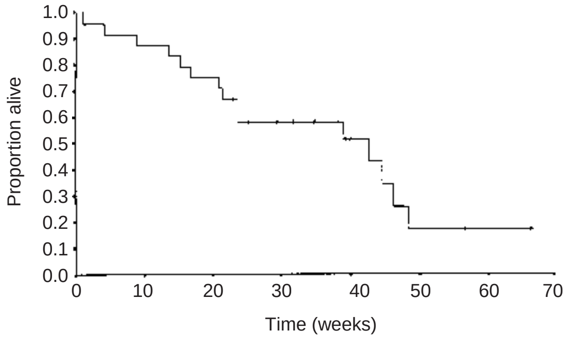

**Figure 1** Kaplan-Meier Survival curve following treatment with ECT.

(95% confidence interval (CI) 21–55%) based on all patients entered into the study.

Three patients were ineligible for response assessment; one patient died from sepsis and gastro-intestinal bleeding after the first cycle of treatment, one patient had treatment discontinued due to grade IV diarrhoea after the first cycle and a further patient had treatment discontinued after one cycle due to early clinical deterioration.

At the time of writing, 15 patients have died. The median follow-up for the nine patients still alive is 8.1 months (range 5.3–15.2 months). The median survival after study entry was 9.9 months (range 0.7–15.2 months). The estimated survival at 6 months is 58% (95% CI 38–77%) (Figure 1).

### DISCUSSION

The recommended dose of raltitrexed in combination with epirubicin and cisplatin for further evaluation is 2.5 mg $m^{-2}$. At this dose level, six patients (50%) completed six cycles and five patients (42%) completed at least three cycles of treatment. Toxicity was mild, although one patient developed grade IV diarrhoea after three cycles of treatment, and there was no evidence of cumulative haematological toxicity. Three patients experienced a mild elevation in hepatic transaminases. This is a recognized toxicity for tomudex, which is asymptomatic, and is only rarely dose-limiting (Clarke et al, 1996). The DLT for this combination is neutropaenia, diarrhoea and stomatitis.

Recent pharmacokinetic studies suggest that up to 60% of raltitrexed clearance is accounted for by renal excretion (Clarke et al, 1996; Beale et al, 1998). It is therefore of interest that renal toxicity with the combination of raltitrexed and cisplatin was minimal. The use of the Cockroft and Gault formula to calculate of creatinine clearance has recently been criticized, especially where this is the basis for calculation of carboplatin dosage according to AUC, as it tends to underestimate creatinine clearance compared to other methods such as inulin clearance and 24-h urinary creatinine clearance (Fliser et al, 1999). Consequently it was considered that this method of calculating creatinine clearance was adequate to assess renal function prior to each cycle of treatment, as the purpose of this assessment was to determine a level of renal function below which dose modification should be performed to prevent toxicity.

Clinical data regarding the use of raltitrexed in gastric cancer are scarce. A single study has demonstrated no activity (Meropol et al, 1996). However more than 50% of patients in that study had received previous 5-FU-based chemotherapy. It is known that the mechanisms of resistance to raltitrexed in vitro include reduced poly-glutamation of raltitrexed and increased expression of TS (Jackman et al, 1995). Increased TS expression may also be induced by 5-FU and correlates with reduced response rate to 5-FU-based treatment. It is also associated with a poor prognosis in patients with gastric cancer (Johnston et al, 1995; Lenz et al, 1996; Boku et al, 1998; Metzger et al, 1998; Yeh et al., 1998). The level of expression of TS is likely to be a clinically relevant predictor for response to raltitrexed and may explain the lack of response seen in the above phase II study, given the number of patients who had received previous 5-FU-based chemotherapy.

Laboratory studies, in ovarian cancer cell lines, suggest that the cytotoxic effects of raltitrexed and cisplatin are at least additive and may be synergistic (Kelland et al, 1995). It is therefore possible that raltitrexed in combination with cisplatin in the treatment of gastric cancer may result in improved response rates compared to either agent on its own, in the absence of preclinical data to suggest antagonism.

The purpose of this study was to define the optimal dose of raltitrexed in combination with epirubicin and cisplatin for use in further studies. Nonetheless, this combination is clearly active in patients with advanced gastro-oesophageal cancer with an overall response rate of 38% in a limited number of evaluable patients. Whilst this is lower than the phase II response rates initially obtained with infusional 5-FU in combination with epirubicin and cisplatin (Findlay et al, 1994; Zaniboni et al, 1995), it is similar to that obtained with other platinum-containing regimens (Elliott et al, 1990; Sparano et al, 1990; Lacave et al, 1991; Cervantes et al, 1993; Bajetta et al, 1994, 1998; Taal et al, 1994; Kondo et al, 1996; Cheng et al, 1998) or with ECF in randomized studies (Webb et al, 1997). The median survival of 9.9 months compares with 8.9 months obtained with ECF and between 7 and 11 months with other cisplatin-based therapies (Lacave et al, 1991; Cervantes et al, 1993; Bajetta et al, 1999, 1998; Kondo et al, 1996; Cheng et al, 1998). We therefore conclude that the optimal dose of raltitrexed is 2.5 mg $m^{-2}$ when used in combination with epirubicin and cisplatin. A multi-centre phase II study of this regimen is in progress with the aim of determining the activity of this regimen in oesophagogastric cancer. If this demonstrates a response rate of greater than 40% with sufficient confidence, a randomized phase III comparison with ECF would be appropriate.

### ACKNOWLEDGEMENTS

'Tomudex' is a trade name, the property of Zeneca Limited. This trial was supported by a grant from Zeneca Pharma, Macclesfield, UK. The authors are grateful to the clinicians who referred patients for this study, and to all the medical and nursing staff who were involved in the care of the patients.

### REFERENCES

Allum WH, Powell DJ, McConkey CC and Fielding JW (1989) Gastric cancer: a 25-year review. *Br J Surg* **76**: 535–540

Bajetta E, Di Bartolomeo M, de Braud F, Bozzetti F, Bochicchio AM, Comella P, Fagnani D, Farina G, Ferroni C and Franchi R (1994) Etoposide, doxorubicin and cisplatin (EAP) treatment in advanced gastric carcinoma: a multicentre study of the Italian Trials in Medical Oncology (I.T.M.O.) Group. *Eur J Cancer* **30A**: 596–600

Bajetta E, Di Bartolomeo M, Carnaghi C, Buzzoni R, Mariani L, Gebbia V, Comella G, Pinotti G, Ianniello G, Schieppati G, Bochicchio AM and Maiorino L (1998) FEP regimen (epidoxorubicin, etoposide and cisplatin) in advanced

© 2000 Cancer Research Campaign gastric cancer, with or without low-dose GM-CSF: an Italian Trial in Medical Oncology (ITMO) study. *Br J Cancer* **77**: 1149–1154

Beale P, Judson I, Hanwell J, Berry C, Aherne W, Hickish T, Martin P and Walker M (1998) Metabolism, excretion and pharmacokinetics of a single dose of [14C]-raltitrexed in cancer patients. *Cancer Chemother Pharmacol*, **42**: 71–76

Boku N, Chin K, Hosokawa K, Ohtsu A, Tajiri H, Yoshida S, Yamao T, Kondo H, Shirao K, Shimada Y, Saito D, Hasebe T, Mukai K, Seki S, Saito H and Johnston PG (1998) Biological markers as a predictor for response and prognosis of unresectable gastric cancer patients treated with 5-fluorouracil and cis-platinum. *Clin Cancer Res* **4**: 1469–1474

Cervantes A, Villar-Grimalt A, Abad A, Anton-Torres A, Belon J, Dorta J, Tres A, Camps C, Fonseca E and Massuti B (1993) 5-Fluorouracil, folinic acid, epidoxorubicin and cisplatin (FLEP) combination chemotherapy in advanced measurable gastric cancer. A phase II trial of the Spanish Cooperative Group for Gastrointestinal Tumor Therapy (TTD). *Ann Oncol* **4**: 753–757

Cheng AL, Yeh KH, Lin JT, Hsu C and Liu MY (1998) Cisplatin, etoposide, and weekly high-dose 5-fluorouracil and leucovorin infusion (PE-HDFL)-a very effective regimen with good patients' compliance for advanced gastric cancer. *Anticancer Res* **18**: 1267–1272

Clarke SJ, Hanwell J, de Boer M, Planting A, Verweij J, Walker M, Smith R, Jackman AL, Hughes LR, Harrap KR, Kennealey GT and Judson IR (1996) Phase I trial of ZD 1694, a new folate-based thymidylate synthase inhibitor, in patients with solid tumours. *J Clin Oncol* **14**: 1495–1503

Cunningham D (1998) Mature results from three large controlled studies with raltitrexed ('Tomudex'). *Br J Cancer* **77**: 15–21

Elliott TE, Moertel CG, Wieand HS, Hahn RG, Gerstner JB, Tschetter LK and Mailliard JA (1990) A phase II study of the combination of etoposide and cisplatin in the therapy of advanced gastric cancer. *Cancer* **65**: 1491–1494

Findlay M, Cunningham D, Norman A, Mansi J, Nicolson M, Hickish T, Nicolson V, Nash A, Sacks N, Ford H and et al (1994) A phase II study in advanced gastro-esophageal cancer using epirubicin and cisplatin in combination with continuous infusion 5-fluorouracil (ECF). *Ann Oncol* **5**: 609–616

Fliser D, Bischoff I, Hanses A, Block S, Joest M, Ritz E and Mutschler E (1999) Renal handling of drugs in the elderly. Creatinine clearance underestimates renal function and pharmacokinetics remain virtually unchanged. *Eur J Clin Pharm* **55**: 205–211

Gore ME, Earl HM, Cassidy J, Tattersall M, Mansi J, Seymour L and Azab M (1995) A phase II study of Tomudex in relapsed epithelial ovarian cancer. *Ann Oncol* **6**: 724–725

Highley MS, Parnis FX, Trotter GA, Houston SJ, Penson RT, Harper PG and Mason RC. Combination chemotherapy with epirubicin, cisplatin and 5-fluorouracil for the palliation of advanced gastric and oesophageal adenocarcinoma. *Br J Surg* **81**: 1763–1765

Jackman AL, Kelland LR, Kimbell R, Brown M, Gibson W, Aherne GW, Hardcastle A and Boyle FT (1995) Mechanisms of acquired resistance to the quinazoline thymidylate synthase inhibitor ZD 1694 (Tomudex) in one mouse and three human cell lines. *Br J Cancer* **71**: 914–924

Johnston PG, Lenz HJ, Leichman CG, Danenberg KD, Allegra CJ, Danenberg PV and Leichman L (1995) Thymidylate synthase gene and protein expression correlate and are associated with response to 5-fluorouracil in human colorectal and gastric tumours. *Cancer Res* **55**: 1407–1412

Kelland LR, Kimbell R, Hardcastle A, Aherne GW and Jackman AL (1995) Relationships between resistance to cisplatin and antifolates in sensitive and resistant tumour cell lines. *Eur J Cancer* **31A**: 981–986

Kondo K, Murase M, Kodera Y, Akiyama S, Ito K, Yokoyama Y, Takagi H and Shirasaka T (1996) Feasibility study on protracted infusional 5-fluorouracil and consecutive low-dose cisplatin for advanced gastric cancer. *Oncology* **53**: 64–67

Lacave AJ, Baron FJ, Anton LM, Estrada E, De Sande LM, Palacio I, Esteban E, Gracia JM, Buesa JM, Fernandez OA and et al (1991) Combination chemotherapy with cisplatin and 5-fluorouracil 5-day infusion in the therapy of advanced gastric cancer: a phase II trial. *Ann Oncol* **2**: 751–754

Lenz HJ, Leichman CG, Danenberg KD, Danenberg PV, Groshen S, Cohen H, Laine L, Crookes P, Silberman H, Baranda J, Garcia Y, Li J and Leichman L (1996) Thymidylate synthase mRNA level in adenocarcinoma of the stomach: a predictor for primary tumour response and overall survival. *J Clin Oncol* **14**: 176–182

Meropol NJ, Pazdur R, Vincent M, Willson JK, Kelsen DP and Douglass HO, Jr (1996) Phase II study of ZD 1694 in patients with advanced gastric cancer. *Am J Clin Oncol* **19**: 628–630

Metzger R, Leichmann CG, Danenberg KD, Danenberg PV, Lenz HJ, Hayashi K, Groshen S, Salonga D, Cohen H, Laine L, Crookes P, Silberman H, Baranda J, Konda B and Leichman L (1998) ERCC1 mRNA levels complement thymidylate synthase mRNA levels in predicting response and survival for gastric cancer patients receiving compbination cisplatin and fluorouracil chemotherapy. *J Clin Oncol* **16**: 309–316

Pazdur R, Meropol NJ, Casper ES, Fuchs C, Douglass HO Jr, Vincent M and Abbruzzese JL (1996) Phase II trial of ZD 1694 (Tomudex) in patients with advanced pancreatic cancer. *Investigat New Drugs* **13**: 355–358

Rigg A, Cunningham D, Gore M, Hill M, O'Brien M, Nicolson M, Chang J, Watson M, Norman A, Hill A, Oates J, Moore H and Ross P (1997) A phase I/II study of leucovorin, carboplatin and 5-fluorouracil (LCF) in patients with carcinoma of unknown primary site or advanced oesophagogastric/pancreatic adenocarcinomas. *Br J Cancer* **75**: 101–105

Smith I, Jones A, Spielmann M, Namer M, Green MD, Bonneterre J, Wander HE, Hatschek T, Wilking N, Zalcberg J, Spiers J and Seymour L (1996) A phase II study in advanced breast cancer: ZD 1694 ('Tomudex') a novel direct and specific thymidylate synthase inhibitor. *Br J Cancer* **74**: 479–481

Sparano JA, Schwartz EL, Salva KM, Pizzillo MF, Wadler S and Wiernik PH (1990) Phase II trial of etoposide, doxorubicin (Adriamycin), and cisplatin (EAP regimen) in advanced gastric cancer. *Am J Clin Oncol* **13**: 374–378

Taal BG, Teller FG, ten Bokkel Huinink WW, Boot H, Beijnen JH and Dubbelman R (1994) Etoposide, leucovorin, 5-fluorouracil (ELF) combination chemotherapy for advanced gastric cancer: experience with two treatment schedules incorporating intravenous or oral etoposide. *Ann Oncol* **5**: 90–92

Webb A, Cunningham D, Scarffe JH, Harper P, Norman A, Joffe JK, Hughes M, Mansi J, Findlay M, Hill A, Oates J, Nicolson M, Hickish T, O'Brien M, Iveson T, Watson M, Underhill C, Wardley A and Meehan M (1997) Randomized trial comparing epirubicin, cisplatin, and fluorouracil versus fluorouracil, doxorubicin, and methotrexate in advanced esophagogastric cancer. *J Clin Oncol* **15**: 261–267

Yeh KH, Shunm CT, Chen CL, Lin JT, Lee WJ, Lee PH, Chen YC and Cheng AL (1998) High expression of thymidylate synthase is associated with drug resistance of gastric carcinoma to high dose 5-fluorouracil based chemotherapy. *Cancer* **82**: 1626–1631

Zaniboni A, Barni S, Labianca R, Marini G, Pancera G, Giaccon G, Piazza E, Signaroldi A, Legnani W and Luporini G (1995) Epirubicin, cisplatin, and continuous infusion 5-fluorouracil is an active and safe regimen for patients with advanced gastric cancer. An Italian Group for the Study of Digestive Tract Cancer (GISCAD) report. *Cancer* **76**: 1694–1699.

*© 2000 Cancer Research Campaign*
